# Beyond Physical Sequelae: Job Satisfaction after Occupational Injuries

**DOI:** 10.1192/j.eurpsy.2026.11086

**Published:** 2026-07-31

**Authors:** A. Haddar, S. Imen, H. Anouare, G. Mohamed Anis, Z. Houcem, F. Afef, T. Moez, M. Mohamed Larbi, H. J. Kaouthar, H. Mounira

**Affiliations:** 1 Occupational Medicine, University Hospital Hedi Chaker; 2 Orthopedics, University Hospital Habib Bourguiba Sfax; 3 Rheumatology, University Hospital Hedi Chaker; 4 Orthopedics, University Hospital Habib Bourguiba, Sfax, Tunisia

## Abstract

**Introduction:**

Occupational injuries remain a major concern in occupational health, as they may compromise both physical recovery and professional reintegration. Job satisfaction is a key determinant of return-to-work and long-term employability.

**Objectives:**

The aim of this study was to evaluated the psychological outcomes of work-related hand injury among private sector employees

**Methods:**

A cross-sectional study was conducted between January 2021 and December 2022 among private sector employees who had sustained work-related hand injuries. Data collection relied on a structured questionnaire addressing sociodemographic and occupational characteristics, details of the occupational injury and relevant medical information. Job satisfaction was assessed using a 0–10 visual analog scale (VAS). Disability was measured using the QuickDASH, and psychological distress using the Kessler Psychological Distress Scale (K6).

**Results:**

Seventy-nine injured workers were included, with a sex ratio (Male/Female) of 3.9. The mean age was 41.3 ± 11.2 years. Current smoking was reported by 38% and alcohol use by 9% of participants. Median smoking exposure was 10 pack-years [IQR 4–20]. The mean QuickDASH score was 34.2 ± 23.2. The mean K6 score was 9.3 ± 6.4, with 31.6% of participants experiencing a high level of psychological distress. The mean job satisfaction score was 5.8 ± 2.5. Job satisfaction was negatively correlated with functional limitation (QuickDASH, r = –0.236, p = 0.039), psychological distress (K6, r = –0.347, p = 0.002), and smoking exposure (pack-years, r = –0.362, p = 0.025).

**Conclusions:**

Job satisfaction after occupational hand injuries was moderate and mainly influenced by functional limitations, psychological distress, and smoking exposure. These findings underline the need for rehabilitation programs that incorporate psychosocial support, smoking cessation, and workplace adaptations to promote job satisfaction and sustainable return-to-work.

**Disclosure of Interest:**

None Declared

